# Predictive Factors Aiding in the Estimation of Intraoperative Resources in Gastric Cancer Oncologic Surgery

**DOI:** 10.3390/cancers17122038

**Published:** 2025-06-18

**Authors:** Alexandru Blidișel, Mihai-Cătălin Roșu, Andreea-Adriana Neamțu, Bogdan Dan Totolici, Răzvan-Ovidiu Pop-Moldovan, Andrei Ardelean, Valentin-Cristian Iovin, Ionuț Flaviu Faur, Cristina Adriana Dehelean, Sorin Adalbert Dema, Carmen Neamțu

**Affiliations:** 1 Department of Surgery I–Clinic of Surgical Semiotics & Thoracic Surgery, Center for Hepato-Biliary and Pancreatic Surgery, “Victor Babes” University of Medicine and Pharmacy, Eftimie Murgu Square, No. 2, 300041 Timisoara, Romania; blidy@umft.ro (A.B.); valentin.iovin@umft.ro (V.-C.I.); 2 Clinical County Emergency Hospital of Arad, Andrenyi Karoly Str., No. 2-4, 310037 Arad, Romania; mihai.roshu@yahoo.ro (M.-C.R.); andreiardelean1986@gmail.com (A.A.); neamtu.carmen@uvvg.ro (C.N.); 3 Faculty of Medicine, “Vasile Goldis” Western University of Arad, Liviu Rebreanu Str., No. 86, 310045 Arad, Romania; 4 Doctoral School of Medicine, “Vasile Goldis” Western University of Arad, Liviu Rebreanu Str., No. 86, 310045 Arad, Romania; flaviu.faur@umft.ro; 5 Department of Toxicology, “Victor Babes” University of Medicine and Pharmacy, Eftimie Murgu Square, No. 2, 300041 Timisoara, Romania; cadehelean@umft.ro; 6 Research Centre for Pharmaco-Toxicological Evaluation, “Victor Babes” University of Medicine and Pharmacy, Eftimie Murgu Square, No. 2, 300041 Timisoara, Romania; 7 “Pius Brinzeu” Clinical County Emergency Hospital of Timisoara, 300723 Timisoara, Romania; razvan.popmoldovan@yahoo.ro; 8 Doctoral School Department, “Victor Babes” University of Medicine and Pharmacy, Eftimie Murgu Square, No. 2, 300041 Timisoara, Romania; 9 X Department of General Surgery, “Victor Babes” University of Medicine and Pharmacy, Eftimie Murgu Square, No. 2, 300041 Timisoara, Romania; 10 Department of Oncology, “Victor Babes” University of Medicine and Pharmacy, Eftimie Murgu Square, No. 2, 300041 Timisoara, Romania; sorin.dema@umft.ro; 11 Department of Radiotherapy, Emergency City Hospital, 300595 Timisoara, Romania; 12 Faculty of Dentistry, “Vasile Goldis” Western University of Arad, Liviu Rebreanu Str., No. 86, 310045 Arad, Romania

**Keywords:** gastric cancer, surgery duration, operating room management, preoperative evaluation, TNM staging, histological grade, lymph node dissection, curative surgery, palliative surgery

## Abstract

**Background/Objectives:** Operating rooms represent valuable and pivotal units of any hospital. Therefore, their management affects healthcare service delivery through rescheduling, staff shortage/overtime, cost inefficiency, and patient dissatisfaction, among others. To optimize scheduling, we aim to assess preoperative evaluation criteria that influence the prediction of surgery duration for gastric cancer (GC) patients. In GC, radical surgery with curative intent is the ideal treatment. Nevertheless, the intervention sometimes must be palliative if the patient’s status and tumor staging prove too advanced. **Methods:** A 6-year retrospective cohort study was performed in a tertiary care hospital, including all cases diagnosed with GC (ICD-10 code C16), confirmed through histopathology, and undergoing surgical treatment (N = 108). **Results:** The results of our study confirm male predominance (63.89%) among GC surgery candidates while bringing new perspectives on patient evaluation criteria and choice of surgical intervention (curative—Group 1, palliative—Group 2). Surgery duration, including anesthesiology (175.19 [95% CI (157.60–192.77)] min), shows a direct correlation with the number of lymph nodes dissected (*Surgical duration [min] = 10.67 × No. of lymph nodes removed − 32.25*). Interestingly, the aggressiveness of the tumor based on histological grade (highly differentiated being generally less aggressive than poorly differentiated) shows differential correlation with surgery duration among curative and palliative surgery candidates. Similarly, TNM staging indicates the need for a longer surgical duration (pTNM stage IIA, IIB, and IIIA) for curative interventions in patients with less advanced stages, as opposed to shorter surgery duration for palliative interventions (pTNM stage IIIC and IV). **Conclusions:** The study quantitatively presents the resources needed for the optimal surgical treatment of different groups of GC patients, as the disease coding systems in use regard the treatment of each pathology as “standard” in terms of patient management. The results obtained are anchored in the global perspectives of surgical outcomes and aim to improve the management of operating room scheduling, staff, and resources.

## Simple Summary

Operating rooms are essential to any hospital, and their efficient management is crucial for ensuring high-quality patient care. When schedules are disrupted, it can lead to delays, staff overwork, increased costs, and dissatisfaction for both patients and healthcare providers. Gastric cancer is a complex disease, and surgery represents the most effective treatment, though the approach may vary depending on the patient’s condition and cancer stage. This study focuses on understanding how preoperative factors, such as blood tests, tumor type, and cancer stage, influence the time needed for surgery. By identifying these factors, we aim to improve the planning and allocation of operating room resources. The findings of this research can help healthcare professionals optimize surgical schedules, reduce unnecessary delays, and ensure better use of hospital resources, ultimately benefiting both patients and medical staff while providing new insights for the broader research community.

## 1. Introduction

The management of operating rooms (ORs) is a complex process, and mismanagement in this area can significantly impact hospital expenses and the quality of care provided at various levels [1,2]. For instance, it can lead to surgery cancellations [3], staff shortage/overtime due to conflicts in planning [4,5], and lack of work satisfaction among healthcare providers [5], among others. From the patient’s perspective, inaccurate scheduling contributes to prolonged waiting times for surgical treatment and heightened stress and anxiety, alongside disease progression resulting from surgery delays [6]. While some of the inconveniences could be overlooked, preoperative stress and anxiety were observed to worsen the surgical outcomes of patients [7], making OR scheduling and management directly related to the well-being of the patients. Consequently, optimizing OR management and efficiency should be a critical priority for many hospitals.

Nevertheless, all surgical procedures, including cancer surgeries, necessitate the passage of an irreversible period of time to be carefully completed. In order to avoid technical failures and human errors, no scheduling conflicts and OR management issues shall concern the surgeons during the intervention [8,9,10]. Performing the procedure hastily increases the risk of causing fatal tissue damage during surgery. One of the aspects of utmost importance during oncologic surgeries is lymphatic dissection, a critical prognostic factor [11,12], which can be a time-intensive process due to the intricate topography of lymphatic drainage [10,12,13]. Conversely, prolonged operative time has been reported to negatively affect surgical outcomes. It is linked to increased intraoperative bleeding, heightened inflammation, and delayed wound healing accompanied by surgical site infections [14,15,16]. The risk of pulmonary complications can be elevated by extended recovery of respiratory function [17,18]. Additionally, increased interstitial fluid accumulation can be caused by the reduced intravascular oncotic pressure [19]. These complications can delay the initiation of a postoperative diet and hospital discharge, contributing to poor nutritional status and weight loss, particularly in gastrointestinal surgeries [20,21,22,23,24,25,26]. As a result, OR management must plan an adequate amount of time for the surgeon to meticulously carry out the procedure [27].

According to GLOBOCAN 2022, gastric cancer (GC) represents the fifth most common malignancy and the fourth leading cause of cancer-related death globally, in spite of the declining trend of incidence and mortality in various countries over the past few decades [28]. In Romania, GC occupies the sixth place in both incidence and mortality among malignancies, with higher rates in male patients [29]. It occurs predominantly in older age groups, with a peak incidence in the sixth decade of life [30]. With human life expectancy increasing nowadays, the proportion of elderly GC patients is also on the rise [31], expected to increase by approximately 30–35% by 2050, according to GLOBOCAN 2022 predictions for Europe [32].

The only course of treatment with curative intent for GC is represented by radical surgical resection as part of a multimodal therapy, despite advances in oncology and radiotherapy, which indicate that neoadjuvant and adjuvant chemotherapy could improve surgical outcomes [33]. With the introduction of neoadjuvant chemotherapy protocols in advanced gastric cancer in the last decade [34], the timing of the surgery should also be correlated with the resolution of related toxicity, improvement of nutritional status, or management of serious comorbidities, aiming to increase patients’ overall time of survival [35,36]. Assessment of criteria for standardization of surgical treatment of GC, tailored to the patient’s specific profile, is vital in the improvement of the outcomes [34]. Nevertheless, the estimation of surgical duration has not yet, to the best of our knowledge, been formally addressed in the scientific literature. This leads to heuristic estimations of intraoperative resources, rather than evidence-based approaches. The current study aims to bridge this gap in the field in order to allow for better OR management.

It can be hypothesized that the GC surgical duration can vary based on factors such as the patient’s preoperative status, the extent of the disease, the type of surgical intervention, and the surgeon’s experience. Based on the current context in oncologic surgery, this article aims to improve the management of OR time and staff through appropriate resource allocation for the benefit of the patient. For this, several preoperative characteristics of GC patients feasible to obtain prior to surgery in transitioning countries are assessed, aiming to lay the basis of a predictive model for calculating the duration of a surgery. The ideal estimation shall be easily accessible to medical doctors and provide underlying principles adaptable to the empirical data of varied surgical oncology teams.

## 2. Materials and Methods

The retrospective cohort study was performed in a tertiary care hospital (the Clinical County Emergency Hospital of Arad, Romania), with a reference population of 410,143 individuals [37], situated in the western part of Romania. All cases diagnosed with GC (ICD-10 code C16) undergoing surgical treatment through the Clinic General Surgery I Department of the Clinical County Emergency Hospital of Arad in the period 1 January 2019–31 December 2024 were considered for inclusion in the study.

Inclusion criteria:

- Patients diagnosed with malignant gastric adenocarcinomas (ICD-10 code C16), confirmed with histopathological diagnosis, who underwent a gastrectomy during the study period in the study center;
- As per institutional policy, only patients with documented general consent for secondary use of data (for research and education) were included.

Exclusion criteria:

- Patients suffering from benign gastric tumors;
- Patients suffering from gastric inflammations (e.g., gastritis);
- Patients suffering from gastric infections (e.g., gastric actinomycosis);
- Patients with a major contraindication for surgery due to the general anesthesia;
- Patients who have explicitly declined the use of their medical data for research and/or medical education purposes, as documented by their non-consent in the institutional records.

The patients were divided into two study groups:

- Group 1: Patients undergoing surgery with curative intent;
- Group 2: Patients undergoing surgery with palliative intent.

The study was conducted in accordance with the Declaration of Helsinki and bears the approval of the Ethics Committee of the Clinical County Emergency Hospital of Arad, Romania (Protocol No. 48 approved on 19 February 2020, revisited for the extension of the duration of the study on 7 March 2025) and the approval of the Ethics and Scientific Research Committee of the Western University “Vasile Goldiș” of Arad, Romania (Protocol No. 10 approved on 21 February 2020). Informed consent for anonymized, unidentifiable secondary patient data usage for clinical research and publication of the data was signed by all study participants at the time of hospital admission. No identifiers are disclosed through the publication of this study.

Statistical analysis was conducted using GraphPad Prism Version 9.5.1 (528), 24 January 2023, for macOS (USA). For comparisons of quantitative data between two groups, an unpaired *t*-test was used with the appropriate Mann–Whitney corrections for non-Gaussian data distribution (also called Mann–Whitney U test), with the level of statistical significance set at alpha = 0.05. Results were considered statistically significant at a *p*-value < 0.05, denoted by an asterisk (*), a *p*-value < 0.01 was denoted by two asterisks (**), a *p*-value < 0.001 by three asterisks (***), and a *p*-value < 0.0001 by four asterisks (****). For the analysis of correlational data, Pearson correlation coefficients were calculated to assess the strength and direction of the linear relationships between variables. Subsequent simple linear regression analysis was performed to fit the data points and derive the regression line that best described the relationship. The specific parameters used for each test, including assumptions checked, such as normality and homogeneity of variances, were recorded to ensure reproducibility and integrity of the analysis.

## 3. Results

**Figure 1:**
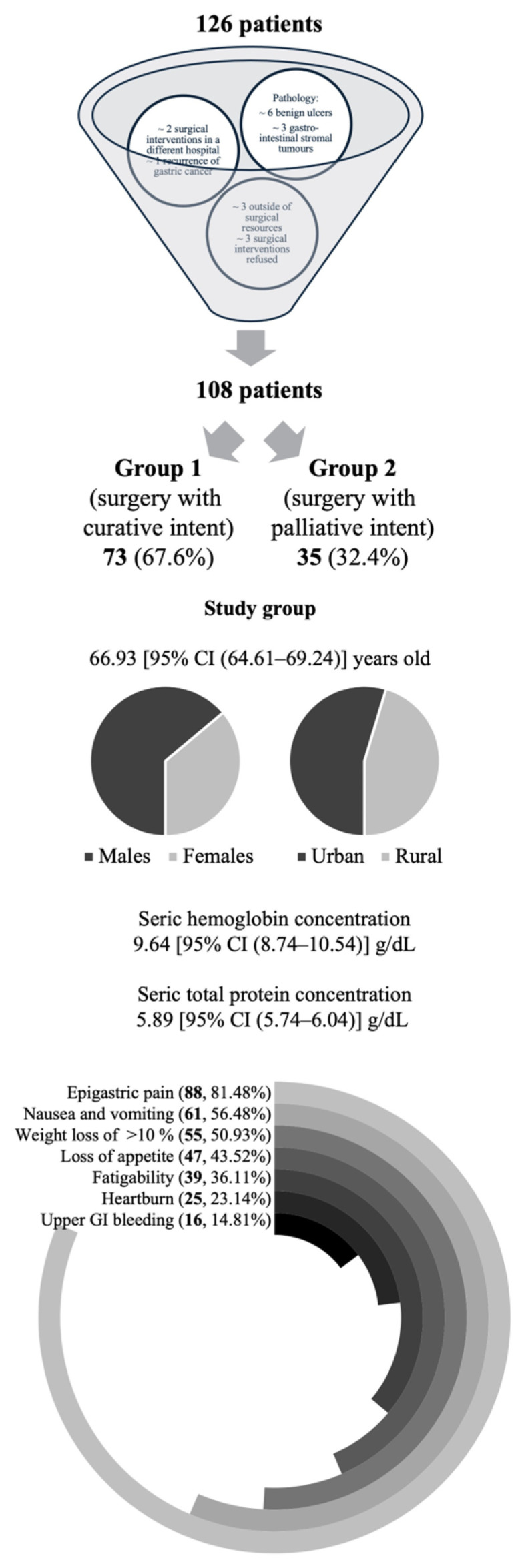
A general overview of the characteristics of the patients included in the study conducted on gastric cancer patients undergoing surgical interventions with curative/palliative intent in the period from 1 January 2019 to 31 December 2024 at the Clinical County Emergency Hospital of Arad, Romania.

In the 6-year period of the study (1 January 2019–31 December 2024), out of the 126 patients admitted with gastric tumors by the Clinic General Surgery I Department of the Clinical County Emergency Hospital of Arad, Romania, 108 patients were included in the study (Figure 1). Nine of the patients were excluded due to histopathological classification as benign ulcerations (six patients) and as gastrointestinal stromal tumors (three patients). Three patients were considered to be outside of surgical resources due to the advanced stage of the disease and patient status deterioration. Three patients refused the surgical intervention. Two patients underwent the gastrectomy in a different hospital. One patient presented with the recurrence of GC.

The average age of the 108 patients included in the study was 66.93 [95% CI (64.61–69.24)] years, with a gender distribution of 69 (63.89%) males and 39 (36.11%) females, and sociodemographic repartition of 59 (54.63%) in the urban and 49 (45.37%) in the rural area. The GC patients included in the study (Figure 1) were admitted by the Surgery Department with the following symptoms: epigastric pain (88, 81.48%); nausea and vomiting (61, 56.48%); weight loss of >10% of the body weight in the last month (55, 50.93%); loss of appetite (47, 43.52%); fatigability (39, 36.11%); heartburn (25, 23.14%); and upper gastrointestinal bleeding (16, 14.81%). The average preoperative serum hemoglobin concentration was 10.41 [95% CI (10.01–10.81)] g/dL, with a minimum of 4.29 g/dL and a maximum of 14.80 g/dL. The average preoperative total serum protein concentration was 5.89 [95% CI (5.74–6.04)] g/dL, with a minimum of 3.69 g/dL and a maximum of 7.46 g/dL.

The average surgical duration among all patients included in the study was 175.19 [95% CI (157.60–192.77)] min. Based on the α = 0.05 significance level, the Spearman correlation test showed that none of the continuous (non-Gaussian distributed) variables analyzed in the study group correlated with the duration of the surgical intervention in a statistically significant manner. Similarly, the Mann–Whitney U test yielded no potential associations between surgical duration and categorical variables.

The topography of the tumors (Table 1) revealed that a majority of tumors were located in the antrum—55 (50.93%) and gastric corpus—31 (28.70%), followed by the pyloric antrum—14 (12.96%). The least common sites were the cardial region—4 (3.70%), and the gastro-esophageal junction and the fundus, observed in 2 (1.85%) patients each. Statistics through ANOVA and unpaired *t*-test with Welch’s correction have been run to assess the difference in surgical duration between GC anatomical localizations. No statistically significant difference was observed at the α = 0.05 significance level. The gastro-esophageal junction and fundus were excluded from the analysis due to low sample size.

Based on the clinical and pathologic TNM staging of the tumors (Table 2), it can be remarked that regionally advanced tumors (stages T_3_ and T_4_) are present in more than 75% of the GC patients included in the study. These tumors fully encapsulate the parietal gastric structure and present a continuous relation with the neighboring organs. It is remarkably unfortunate that only about one-quarter of patients were diagnosed and referred for the oncologic surgical intervention in incipient stages of the pathology (stages T_is_, T_1_, and T_2_).

**Figure 2:**
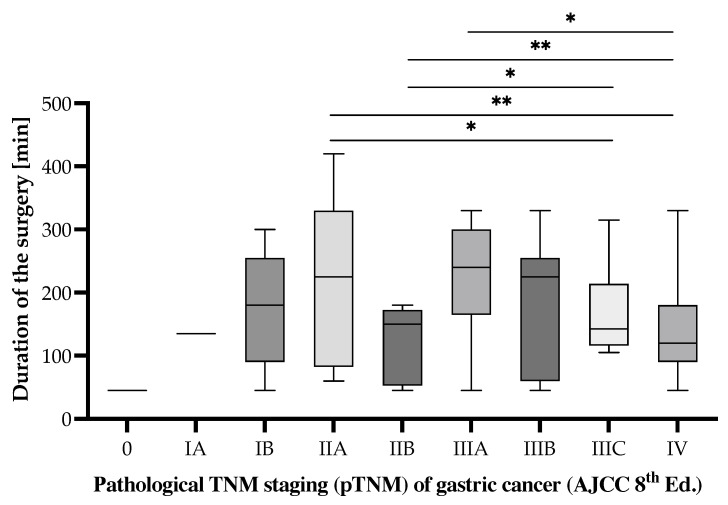
Surgical duration according to pathological TNM staging (pTNM) of the gastric cancer patients undergoing surgical interventions in the period from 1 January 2019 to 31 December 2024 who were included in the study. Statistically significant differences according to unpaired *t*-test for non-Gaussian distributed samples, employing the Mann–Whitney correction. Note: *p* < 0.05 denoted as “*” and *p* < 0.01 denoted as “**”.

According to the pathological TNM staging, there is a statistically significant difference in the duration of surgeries undergone by patients with pTNM staging of IIA, IIB, and IIIA, compared to those with pTNM staging of IIIB and IV (Figure 2). These were identified using the Mann–Whitney U test (*p* < 0.05) between the following stage pairs: IIA vs. IIIC (*p* = 0.0367), IIA vs. IV (*p* = 0.0064), IIB vs. IIIC (*p* = 0.0187), IIB vs. IV (*p* = 0.0039), and IIIA vs. IV (*p* = 0.0141). Under these circumstances, the more advanced the stage of the disease, the less the interventional time required for the surgery.

**Figure 3:**
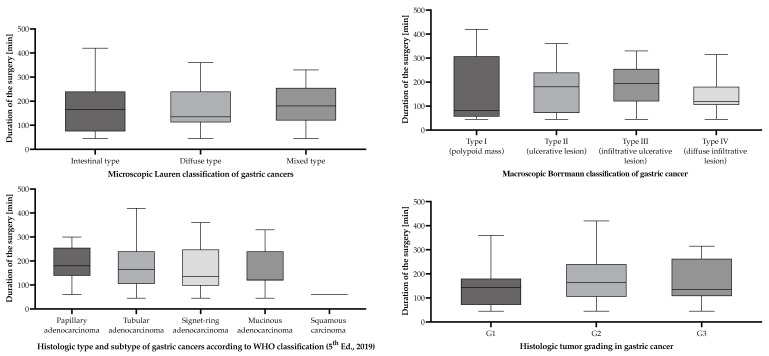
Surgical duration according to the macroscopic Borrmann classification, Lauren classification, WHO histological type, and tumor grading of gastric cancer patients undergoing surgical interventions with curative/palliative intent in the period from 1 January 2019 to 31 December 2024, who were included in the study. Statistically significant differences were not detected between groups.

Among the study group, 76 patients (70.4%) had macroscopic appearance and growth pattern classified as Borrmann III and Borrmann IV, which are generally associated with deeper tissue infiltration and more advanced local disease (Table 3, Figure 3). According to the Lauren classification, GC is divided into three subcategories: intestinal—62 (57.40%), diffuse—21 (19.40%), and mixed—25 (23.10%) patients. Most gastric cancers diagnosed in the study period were adenocarcinomas, except for one squamous carcinoma. The histological type of gastric adenocarcinomas presented most often as a tubular pattern in 73 (67.60%) of the GC patients, followed by mucinous—15 (13.90%) patients, signet-ring—13 (12.00%), and papillary—6 (5.60%) patients. Well-differentiated intestinal type GC was often associated with *Helicobacter pylori* infections and mostly observed among men. On the other hand, advanced diffuse-type GC was less differentiated and was most often diagnosed among women. Tumor grading revealed only 8 (7.40%) tumors to be well differentiated (G1), most moderately differentiated (G2)—87 (80.60%), and 13 (12.00%) poorly differentiated tumors.

**Figure 4:**
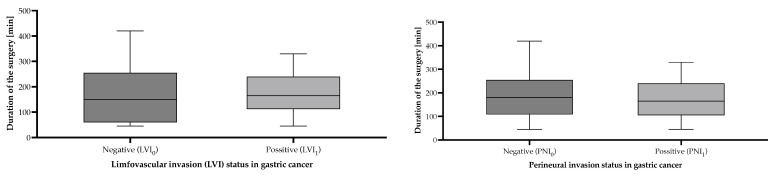
Surgical duration according to lymphovascular and perineural invasion status of gastric cancer patients undergoing surgical interventions with curative/palliative intent in the period from 1 January 2019 to 31 December 2024, who were included in the study. Statistically significant differences were not detected between groups.

Based on the histopathological reports, 73 (67.60%) of the GC patients presented with lymphovascular invasion (LVI_1_) and perineural invasion (PNI_1_) in the primary tumor (Table 4, Figure 4).

**Figure 5:**
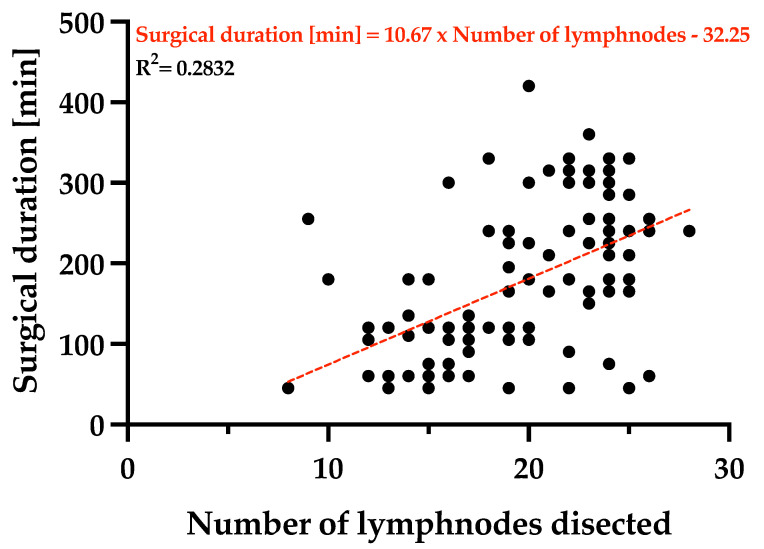
Surgical duration versus the number of lymph nodes dissected from gastric cancer patients undergoing surgical interventions with curative/palliative intent in the period from 1 January 2019 to 31 December 2024, who were included in the study. Spearman correlation test with correlation coefficient of r = 0.54 and *p* < 0.001 indicates a significant association between the surgical duration and the number of lymph nodes removed. A simple linear regression model: *Y = 10.67 × X − 32.25*; R^2^ = 0.2832.

In the present study, a statistically significant positive correlation was observed between the number of lymph nodes dissected and the duration of the surgical intervention (Figure 5). Using the Spearman correlation test, the correlation coefficient was r = 0.54 with a *p* < 0.001, indicating a significant association. This suggests that as the number of lymph nodes removed increases, the surgical duration tends to be longer. These findings are consistent with the clinical expectation that more extensive lymphadenectomy, often required in oncologic gastric surgery for accurate staging and improved local control, contributes to increased operative time.

**Figure 6:**
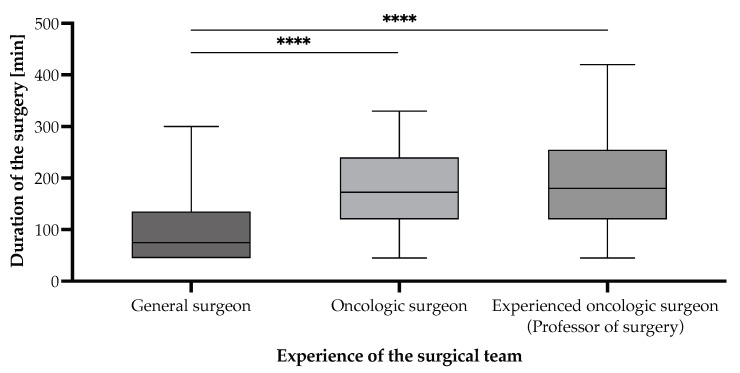
Surgical duration according to the experience of the surgical team of gastric cancer patients undergoing surgical interventions with curative/palliative intent in the period from 1 January 2019 to 31 December 2024, who were included in the study. Statistically significant differences according to the unpaired *t*-test for non-Gaussian distributed samples, employing the Mann–Whitney correction. Note: *p* < 0.0001 denoted as “****”.

**Figure 7:**
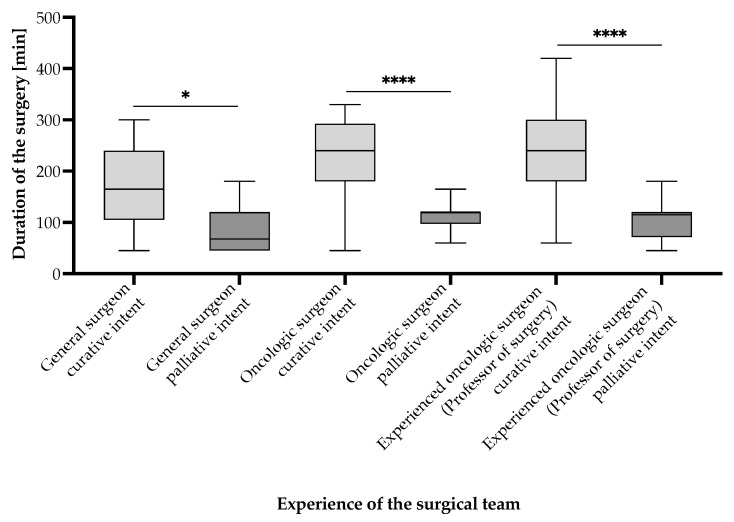
Surgical duration according to the intent of the intervention, alongside the experience of the gastric cancer surgical team for patients undergoing surgical interventions in the period from 1 January 2019 to 31 December 2024, who were included in the study. Statistically significant differences according to the unpaired *t*-test for non-Gaussian distributed samples, employing the Mann–Whitney correction. Note: *p* < 0.05 denoted as “*” and *p* < 0.0001 denoted as “****”.

**Figure 8:**
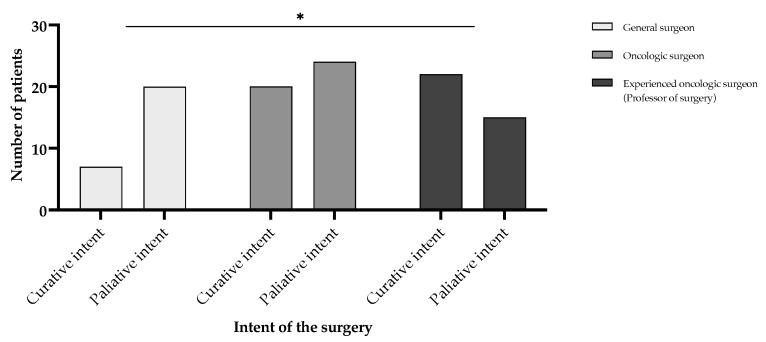
Number of surgeries performed according to the intent of the intervention, alongside the experience of the gastric cancer surgical team for patients undergoing surgical interventions in the period from 1 January 2019 to 31 December 2024, who were included in the study. Statistically significant differences according to the unpaired *t*-test for non-Gaussian distributed samples, employing the Mann–Whitney correction. Note: *p* < 0.05 denoted as “*”.

Surgical duration varied significantly depending on the attending surgeon’s expertise in an unexpected but plausible manner (Figure 6). Procedures performed by general surgeons had the shortest mean duration of 107.20 [95% CI (80.60–133.80)] min, whereas those performed by oncologic surgeons averaged 180.20 [95% CI (159.20–201.20)] min, and those led by experienced oncologic surgeons (professor-level) averaged 198.60 [95% CI (169.10–228.10)] min. Statistical analysis using the Mann–Whitney U test revealed that surgical durations were significantly longer for oncologic surgeons compared to general surgeons (*p* < 0.0001) and for experienced oncologic surgeons compared to general surgeons (*p* < 0.0001). However, there was no significant difference between oncologic and experienced oncologic surgeons (*p* = 0.3808). These results suggest that oncologic expertise is associated with longer operative times, likely reflecting increased procedural complexity and oncologic thoroughness of the cases (Figure 7 and Figure 8). Notably, curative interventions were more frequently performed by oncologic (235.50 [95% CI (197.70–273.30)] min, n = 20, 18.5% patients vs. palliative 148.10 [95% CI (120.70–175.50)] min, n = 24, 22.2% patients) and experienced oncologic surgeons (247.50 [95% CI (216.50–278.50)] min, n = 22, 20.4% patients vs. palliative 107.30 [95% CI (86.30–128.30)] min, n = 15, 13.9% patients), whereas general surgeons predominantly performed palliative procedures (85.50 [95% CI (64.60–106.40)] min, n = 20, 18.5% patients vs. curative 169.30 [95% CI (90.50–248.10)] min, n = 7, 6.5% patients). The longest procedures were consistently observed in curative cases managed by experienced oncologic surgeons (up to 420 min), emphasizing the role of surgical expertise in handling complex oncologic resections. Statistical comparisons of surgical durations between subgroups revealed multiple significant differences (Figure 7). Within general surgeons, curative procedures were significantly longer than palliative ones (*p* = 0.0216). Among oncologic surgeons, curative interventions were also significantly longer than palliative ones, in line with the experienced oncologic surgeon group (*p* < 0.0001). Moreover, there is a statistically significant difference in the distribution of curative and palliative surgeries between general surgeons and experienced oncologic surgeons (*p* = 0.0161), indicating that experienced oncologic surgeons are significantly more likely to perform curative procedures (Figure 8). However, no significant difference was observed between general vs. oncologic surgeons (*p* = 0.1634) or oncologic vs. experienced oncologic surgeons (*p* = 0.3014).

For the comparative analysis of surgical duration, patients included in this retrospective study were divided into two groups, as follows:

- Group 1: Patients undergoing surgery with curative intent—63 (58.33%) patients;
- Group 2: Patients undergoing surgery with palliative intent—45 (32.41%) patients.

**Figure 9:**
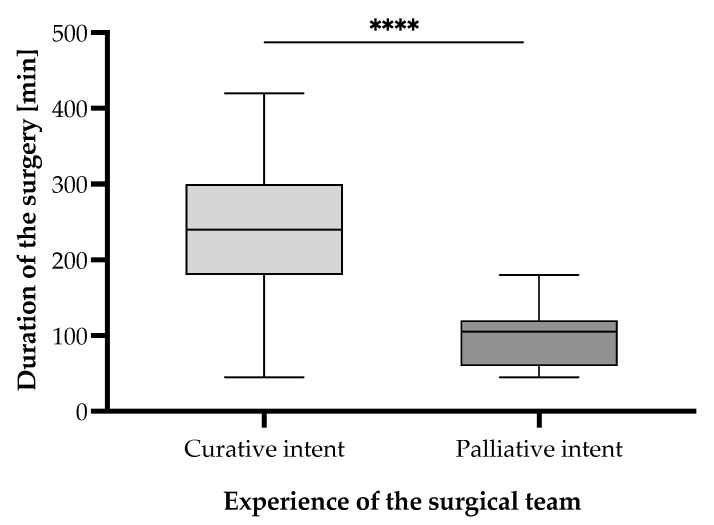
Surgical duration according to the intent of the surgical intervention type—curative (Group 1) or palliative (Group 2)—undergone by the gastric cancer patients in the period from 1 January 2019 to 31 December 2024, who were included in the study. Statistically significant differences according to the unpaired *t*-test for non-Gaussian distributed samples, employing the Mann–Whitney correction. Note: *p* < 0.0001 denoted as “****”.

The 63 patients in Group 1 (surgical intervention with curative intent) can be briefly described as 24 (38.10%) females and 39 (61.90%) males, with an average age of 67.7 [95% CI (64.7–70.7)] years, and an average surgical duration of 227.4 [95% CI (207.7–247.1)] min (Figure 9). An average of 22.1 [95% CI (21.2–22.9)] lymph nodes were dissected in patients in Group 1—with the exception of one patient who was excluded due to pN_x_ status and the lack of dissection—with a minimum of 9 and a maximum of 28 lymph nodes. A correlation was disputed between the number of lymph nodes removed and the duration of the surgical intervention within this group, employing the Spearman correlation test (*p* = 0.051). According to the histologic type, within Group 1, signet-ring (267.5 [95% CI (205.0–330.0)], n = 6, 9.5% patients) and mucinous (252.0 [95% CI (185.6–318.4)], n = 5, 7.9% patients) adenocarcinomas showed a much higher surgical duration than tubular (221.2 [95% CI (196.6–245.7)], n = 47, 74.6% patients) or papillary (213.0 [95% CI (142.8–283.2)], n = 5, 7.9% patients) types (Figure 7). Nevertheless, due to the low number of entries in all categories, with the exception of tubular adenocarcinoma, the statistical analysis could be enhanced by an increased sample size. The Lauren classification shows results aligned with the histologic type.

The 45 patients in Group 2 (surgical intervention with palliative intent) can be briefly described as 14 (31.11%) females and 31 (68.89%) males, with an average age of 65.9 [95% CI (62.1–69.7)] years, and an average surgical duration of 97.8 [95% CI (86.0–109.6)] min (Figure 9). An average of 15.4 [95% CI (14.5–16.3)] lymph nodes were dissected in patients in Group 2—with the exception of 5 patients who were excluded due to pN_x_ status and the lack of dissection—with a minimum of 8 and a maximum of 22 lymph nodes per patient. There was no correlation between the number of lymph nodes removed and the duration of the surgical intervention within this group, employing the Spearman correlation test (*p* = 0.427). According to the histologic type, within Group 2, mucinous (117.0 [95% CI (89.4–144.6)], n = 10, 22.2% patients) adenocarcinoma showed a much higher surgical duration than tubular (94.8 [95% CI (78.7–110.9)], n = 26, 57.8% patients) or signet-ring (92.1 [95% CI (59.6–124.6)], n = 7, 15.6% patients) types; papillary and mucinous type adenocarcinoma, alongside squamous carcinoma, were under-represented within the study group (Figure 7). Nevertheless, due to the low number of entries in all categories, with the exception of tubular adenocarcinoma, the statistical analysis could be enhanced by an increased sample size. The Lauren classification shows results in alignment with the histologic type.

**Figure 10:**
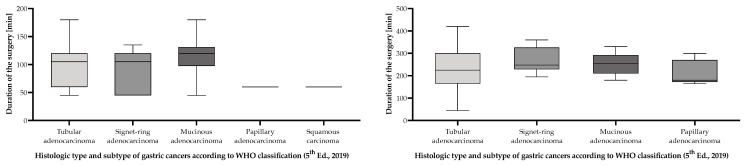
Surgical duration according to the WHO histological type of gastric cancer patients undergoing surgical interventions with curative (Group 1) and palliative (Group 2) intent in the period from 1 January 2019 to 31 December 2024, who were included in the study. Statistically significant differences were not detected between groups.

The comparison of the two study groups clearly reveals a difference of approximately 2 h in between the duration of curative intent surgeries (Group 1), lasting for 227.4 [95% CI (207.7–247.1)] min, and the palliative (Group 2) ones, lasting for 97.8 [95% CI (86.0–109.6)] min (Figure 9 and Figure 10). The difference proves to be statistically significant through the unpaired *t*-test, with a *p* value of *p* < 0.0001.

## 4. Discussion

GC continues to be a major public health issue worldwide, being among the top 10 causes of death associated with neoplasia [36]. Despite real advancement in diagnosis and therapeutic tools improving survival outcomes of GC patients, the complete resection and adequate lymphadenectomy remain the goal of the treatment with curative intent [39,40]. The extent of lymph node dissection remains to date a controversial issue debated among experienced European and Japanese surgeons [41]. Nevertheless, most international surgical committees agree that D2 lymphadenectomy is to be considered the standard of treatment with curative intent for tumors that surpass the T_1b_N_0_ staging according to the AJCC 8th Edition [36,42,43]. Better outcomes have been observed for oncologic surgeries performed in “high volume” hospitals, for which the first minimum threshold was set in 2001 by the Association of Upper Gastrointestinal Surgeons of Great Britain and Ireland, with 15–20 surgical resections per year in the case of GC treatment, which remains the standard according to more recent guidelines [39,44]. Our study center complies with these requirements, with 108 patients undergoing either curative or palliative-intent surgery within six years, an average of approximately 18 annually.

Despite its importance in the management of the OR schedule and patient outcome, to the best of our knowledge, there is no study to date to primarily address the intraoperative duration of radical gastric resections or palliative surgeries of GC patients and their estimation according to preoperative patient evaluation. Nevertheless, some studies estimate the mean surgical duration of total gastrectomy to be about 3 h and that of partial resection to be approximately 2 h for surgeries conducted classically and laparoscopically in the USA [45]. The literature data are in accordance with our study results, averaging the duration of curative intent surgeries (Group 1) at 227.4 [95% CI (207.7–247.1)] min.

According to the scientific literature, it is observed that gastrectomy combined with chemotherapy regimens can improve the survival of patients with stage IV GC, palliative gastrectomy maintaining its importance in relieving symptoms and the treatment of emergent complications [46,47,48]. The average surgical duration for palliative interventions observed in our study was 97.8 [95% CI (86.0–109.6)] min, about 2 h shorter compared to the duration of curative surgery. The main difference in time could be accounted through the lack of appropriate lymph node dissection in palliative procedures—22.1 [95% CI (21.2–22.9)] lymph nodes in Group 1 (curative intent) and 15.4 [95% CI (14.5–16.3)] lymph nodes in Group 2 (palliative intent).

A correlation was observed between the surgical duration and the number of lymph nodes dissected. The correlation is described by the linear regression equation: *Surgical duration [min] = 10.67 × No. of lymph nodes removed − 32.25*, lymph nodes dissected through D1 and D2 dissections. This satisfies, in most cases, the required number of lymph nodes to be examined for the histopathologic staging of the tumoral dissemination [30].

When considering OR scheduling, it is observed that longer surgery durations are necessary for patients undergoing surgeries with curative intent. Nevertheless, scheduling all surgeries for the highest amount of time could create a financial and managerial burden for the hospital and negatively impact access to surgical procedures for patients. With spatial and personnel limitations, it is desirable to estimate more accurately the duration of the surgical intervention based on the preoperative evaluation of the GC patient through parameters offered by endoscopic procedures, imaging and pathologic assessment, and laboratory analyses, constituting the topic of our study.

This retrospective study presents several limitations that should be acknowledged. First, the single-center design and relatively modest sample size (N = 108) may limit the generalizability of findings, particularly in healthcare settings with different surgical practices or patient demographics. The use of retrospective data also introduces potential biases, such as selection bias and incomplete data capture. Additionally, the lack of standardized operative protocols across surgeons with varying experience levels may have introduced variability in surgical durations unrelated to tumor characteristics. Some subgroups—such as specific tumor types and advanced pTNM stages—were under-represented, reducing the statistical power to draw definitive conclusions. Finally, preoperative variables used for prediction did not reach statistical significance in multivariate correlation analyses, indicating that further prospective studies with larger, multicenter cohorts are warranted to validate and refine the proposed predictive models.

Future perspectives involve refining and validating predictive models that estimate surgical duration based on preoperative clinical, pathological, and imaging parameters. These models could be integrated into hospital management systems to enhance operating room scheduling, reduce delays, and improve patient outcomes. Additionally, multicenter prospective studies with larger and more diverse cohorts are essential to confirm the model’s accuracy and applicability across different healthcare settings. Incorporating machine learning algorithms may further enhance predictive precision and support real-time decision-making in surgical oncology.

## 5. Conclusions

The current publication aims to provide an analysis of preoperative characteristics aiding in the estimation of the surgical duration for interventions undergone by gastric cancer patients. The retrospective study was conducted on 108 patients with average age of 66.93 [95% CI (64.61–69.24)] years, with a gender distribution of 63.89% males, and 54.63% participants living in the urban area, presenting for hospital admission with epigastric pain (81.48%), nausea and vomiting (56.48%), and >10% body weight loss in the last month (50.93%). The average preoperative serum hemoglobin concentration was 10.41 [95% CI (10.01–10.81)] g/dL, and the total serum protein concentration was 5.89 [95% CI (5.74–6.04)] g/dL. Tumor topography was mainly classified as antrum (50.93%), gastric corpus (28.70%), and pyloric antrum (12.96%). The surgical duration varied based on the pTNM staging of the tumor, with a statistically significant difference between the duration of surgeries undergone by patients with early staging of IIA, IIB, and IIIA, compared to patients with advanced staging of IIIC and IV. Based on lymph node dissection, the time of the procedure was fitted into the equation: *Surgical duration [min] = 10.67 × Number of lymph nodes − 32.25*, using a simple linear regression model. While counterintuitive, statistically significant longer times were needed by more experienced teams than general surgeons without specialization in oncologic surgery, likely due to the complexity of cases, with experts performing, on average, surgeries lasting double the time. By dividing the patients into two cohorts, based on the curative/palliative intent of the surgery, the curative intent oncologic surgeries lasted an average of 227.4 [95% CI (207.7–247.1)] min, including the dissection of 22.1 [95% CI (21.2–22.9)] lymph nodes per patient. The comparison of the two study groups clearly reveals a difference of approximately 2 h between curative intent surgeries and the palliative ones—97.8 [95% CI (86.0–109.6)] min (*p* < 0.0001). This article aims to encourage the allocation of sufficient time for appropriate lymph node dissection by experienced teams in order to improve patient outcomes and chances of survival, with clear consideration for the importance of OR management and hospital resources.

## Figures and Tables

**Table 1:** A summary of tumor topography presented alongside the average surgical duration (including anesthesiology) for gastric cancer patients undergoing surgical interventions with curative/palliative intent in the period from 1 January 2019 to 31 December 2024, who were included in the study.

Topography of the Tumor	Number of Patients	Percentage of Patients [%]	Average Surgical Duration (Incl. Anesthesiology) [min]
Gastro-esophageal junction	2	1.85	60.00 [CI not available due to low sample size]
Cardia	4	3.70	142.50 [95% CI (6.78–278.22)]
Fundus	2	1.85	180.00 [CI not available due to low sample size]
Corpus	31	28.70	172.26 [95% CI (136.58–207.94)]
Antrum	55	50.93	182.82 [95% CI (157.55–208.09)]
Pyloric antrum	14	12.96	176.79 [95% CI (128.60–224.97)]

**Table 2:** A summary of tumor TNM staging (according to AJCC 8th Edition) presented alongside the average surgical duration (including anesthesiology) for gastric cancer patients undergoing surgical interventions with curative/palliative intent in the period from 1 January 2019 to 31 December 2024, who were included in the study.

TNM Classification for Gastric Cancer (According to AJCC 8th Edition) [38]	Number of Patients	Percentage of Patients [%]	Average Surgical Duration (Incl. Anesthesiology) [min]
***Size and extent of the main tumor***			
**pT_is_**	1	0.93	45.00 [CI not available due to low sample size]
**pT_1_**	2	1.85	112.50 [CI not available due to low sample size]
**pT_2_**	23	21.30	156.1 [95% CI (114.4–197.8)]
**pT_3_**	20	18.52	192.8 [95% CI (146.1–239.4)]
**pT_4a_**	33	30.56	201.4 [95% CI (174.0–228.7)]
**pT_4b_**	29	26.85	150.5 [95% CI (116.6–184.4)]
***Number of nearby lymph nodes invaded by the tumor***			
**pN_0_**	17	15.74	199.4 [95% CI (147.3–251.6)]
**pN_1_**	36	33.33	171.7 [95% CI (139.5–203.9)]
**pN_2_**	25	23.25	174.6 [95% CI (133.9–215.3)]
**pN_3a_**	13	12.04	174.6 [95% CI (125.5–223.7)]
**pN_3b_**	11	10.19	151.4 [95% CI (106.9–195.9)]
**pN_x_**	6	5.56	142.5 [95% CI (66.2–218.8)]
***Presence of metastases***			
**M_0_**	64	59.26	192.42 [95% CI (168.49–216.36)]
**M_1_**	44	40.74	145.68 [95% CI (122.43–168.93)]
***Pathological TNM staging (pTNM)***			
**0**	1	0.93	45 [CI not available due to low sample size]
**IA**	1	0.93	330 [CI not available due to low sample size]
**IB**	7	6.48	182.14 [95% CI (99.13–265.16)]
**IIA**	9	8.33	218.33 [95% CI (119.13–317.54)]
**IIB**	5	4.63	120 [95% CI (48.09–197.91)]
**IIIA**	26	24.07	222.69 [95% CI (189.52–255.86)]
**IIIB**	10	9.26	180 [95% CI (102.13–257.87)]
**IIIC**	6	5.56	167.50 [95% CI (85.76–249.25)]
**IV**	43	39.81	142.09 [95% CI (119.46–164.72)]

**Table 3:** A summary of tumor characteristics, including the macroscopic Borrmann classification, Lauren classification, WHO histological type, and tumor grading, presented alongside the average surgical duration (including anesthesiology) of gastric cancer patients undergoing surgical interventions with curative/palliative intent in the period from 1 January 2019 to 31 December 2024, who were included in the study.

Classification	Number of Patients	Percentage of Patients [%]	Average Surgical Duration (Incl. Anesthesiology) [min]
***Macroscopic Borrmann classification of gastric cancers***			
Borrmann I	10	9.30	154.5 [95% CI (54.2–254.8)]
Borrmann II	22	20.40	173.9 [95% CI (133.2–214.5)]
Borrmann III	47	43.50	190.6 [95% CI (165.3–216.0)]
Borrmann IV	29	26.9	151.6 [95% CI (122.9–180.2)]
***Microscopic Lauren classification of gastric cancers***			
Intestinal	62	57.40	168.7 [95% CI (144.2–193.2)]
Diffuse	21	19.40	163.6 [95% CI (124.2–202.9)]
Mixed	25	23.10	193.2 [95% CI (159.7–226.7)]
***Histologic type and subtype of gastric cancers according to WHO classification (5th Ed., 2019)***			
**Adenocarcinoma**			
Tubular pattern	73	67.60	176.2 [95% CI (154.4–198.0)]
Signet-ring cell pattern	13	12.00	173.1 [95% CI (111.5–234.6)]
Papillary pattern	6	5.60	187.5 [95% CI (103.2–271.8)]
**Mucinous adenocarcinoma**	15	13.90	162.0 [95% CI (118.7–205.3)
**Squamous carcinoma**			
Squamous cell carcinoma	1	0.93	330 [CI not available due to low sample size]
***Microscopic tumor grading of gastric cancers according to the WHO classification (5th Ed., 2019)***			
G1	8	7.40	151.9 [95% CI (69.4–234.4)]
G2	87	80.60	175.3 [95% CI (156.0–194.7)]
G3	13	12.00	173.5 [95% CI (116.6–230.3)]

**Table 4:** Lymphovascular and perineural invasion status presented alongside the average surgical duration (including anesthesiology) of gastric cancer patients undergoing surgical interventions with curative/palliative intent in the period from 1 January 2019 to 31 December 2024, who were included in the study.

Classification	Number of Patients	Percentage of Patients [%]	Average Surgical Duration (Incl. Anesthesiology) [min]
***Lymphovascular invasion status***			
LVI_0_	35	32.40	170.3 [95% CI (133.7–206.9)]
LVI_1_	73	67.60	174.9 [95% CI (155.4–194.3)]
***Perineural invasion status***			
PNI_0_	46	42.60	184.3 [95% CI (155.5–213.2)]
PNI_1_	62	57.40	165.2 [95% CI (143.3–187.1)]

## Data Availability

The original contributions presented in this study are included in the article. Further inquiries can be directed to the corresponding authors.

## References

[1] Gür Ş., Pınarbaşı M., Alakaş H.M., Eren T. (2022). Operating room scheduling with surgical team: A new approach with constraint programming and goal programming. Cent. Eur. J. Oper. Res..

[2] Bellini V., Guzzon M., Bigliardi B., Mordonini M., Filippelli S., Bignami E. (2019). Artificial Intelligence: A New Tool in Operating Room Management. Role of Machine Learning Models in Operating Room Optimization. J. Med. Syst..

[3] Schofield W.N., Rubin G.L., Piza M., Lai Y.Y., Sindhusake D., Fearnside M.R., Klineberg P.L. (2005). Cancellation of operations on the day of intended surgery at a major Australian referral hospital. Med. J. Aust..

[4] Thompson T.P., Brown H.N. (2002). Turnover of licensed nurses in skilled nursing facilities. Nurs. Econ..

[5] Strachota E., Normandin P., O’brien N., Clary M., Krukow B. (2003). Reasons Registered Nurses Leave or Change Employment Status. Jona J. Nurs. Adm..

[6] Armoeyan M., Aarabi A., Akbari L. (2021). The Effects of Surgery Cancellation on Patients, Families, and Staff: A Prospective Cross-Sectional Study. J. PeriAnesth. Nurs..

[7] Han L. (2020). Prevalence, risk factors and prognostic role of anxiety and depression in surgical gastric cancer patients. Transl. Cancer Res..

[8] Dinkel M., Kamp H.D., Schweiger H. (1991). Somatosensory evoked potentials in carotid surgery. Anaesthesist.

[9] Baqué P., Iannelli A., Delotte J., de Peretti F., Bourgeon A. (2008). Division of the right posterior attachments of the head of the pancreas with a linear stapler during pancreaticoduodenectomy: Vascular and oncological considerations based on an anatomical cadaver-based study. Surg. Radiol. Anat..

[10] Ishiyama Y., Maeda C., Shimada S., Kudo S.-E. (2020). Propensity-score-matched analysis of short- and long-term outcomes in patients with an ileocolic artery crossing anterior vs. posterior to the superior mesenteric vein during curative resection for right-sided colon cancer. Surg. Endosc..

[11] Songun I., Putter H., Kranenbarg E.M.-K., Sasako M., van de Velde C.J. (2010). Surgical treatment of gastric cancer: 15-year follow-up results of the randomised nationwide Dutch D1D2 trial. Lancet Oncol..

[12] Freytag D., Pape J., Dhanawat J., Günther V., Maass N., Gitas G., Laganà A.S., Allahqoli L., Meinhold-Heerlein I., Moawad G.N. (2020). Challenges Posed by Embryonic and Anatomical Factors in Systematic Lymphadenectomy for Endometrial Cancer. J. Clin. Med..

[13] Natsume T., Shuto K., Yanagawa N., Akai T., Kawahira H., Hayashi H., Matsubara H. (2010). The classification of anatomic variations in the perigastric vessels by dual-phase CT to reduce intraoperative bleeding during laparoscopic gastrectomy. Surg. Endosc..

[14] Cheng H., Chen B.P., Soleas I.M., Ferko N.C., Cameron C.G., Hinoul P. (2017). Prolonged operative duration increases risk of surgical site infections: A systematic re-view. Surg. Infect..

[15] Chen K.-W., Yang H.-L., Lu J., Wang G.-L., Ji Y.-M., Bao Z.-H., Wu G.-Z., Gu Y., Sun Z.-Y., Zhu R.-F. (2011). Risk Factors for Postoperative Wound Infections of Sacral Chordoma After Surgical Excision. J. Spinal Disord. Tech..

[16] Nandi P.L., Soundara Rajan S., Mak K.C., Chan S.C., So Y.P. (1999). Surgical wound infection. Hong Kong Med. J..

[17] Schricker T., Lattermann R. (2015). Perioperative catabolism. Can. J. Anaesth..

[18] Gu A., Wei C., Chen A.Z., Malahias M.-A., Fassihi S.C., Ast M.P., Liu J., Cross M.B., Sculco P.K. (2020). Operative time greater than 120 minutes is associated with increased pulmonary and thromboembolic complications following revision total hip arthroplasty. Eur. J. Orthop. Surg. Traumatol..

[19] Lucas C.E., Benishek D.J., Ledgerwood A.M. (1982). Reduced Oncotic Pressure After Shock. Arch. Surg..

[20] Wang X., Yao Y., Qian H., Li H., Zhu X. (2019). Longer Operating Time During Gastrectomy Has Adverse Effects on Short-Term Surgical Outcomes. J. Surg. Res..

[21] Kobayashi T., Watanabe Y., Aizawa J., Suzuki K.S. (2017). Factors affecting the early post-operative prognosis in morbidly obese surgical patients after laparoscopic sleeve gastrectomy—A retrospective cohort study. JA Clin. Rep..

[22] Borta S.M., Dumitra S., Miklos I., Popetiu R., Pilat L., Pușchiță M., Marian C. (2020). Clinical Relevance of Plasma Concentrations of MBL in Accordance with IgE Levels in Children Diagnosed with Bronchial Asthma. Medicina.

[23] Muntean C., Blidari A.R., Faur A.M., Curca R.O., Feier C.V.I. (2024). Evaluating the Outcomes in Patients with Colorectal Cancer Using the Malnutrition Universal Screening Tool: A Systematic Review. J. Multidiscip. Healthc..

[24] Kang D., Yoo K.Y. (2019). Fluid management in perioperative and critically ill patients. Acute Crit. Care.

[25] Lim H.-S., Lee B., Cho I., Cho G.S. (2020). Nutritional and Clinical Factors Affecting Weight and Fat-Free Mass Loss after Gastrectomy in Patients with Gastric Cancer. Nutrients.

[26] Knight S.R., Qureshi A.U., Drake T.M., Lapitan M.C.M., Maimbo M., Yenli E., Tabiri S., Ghosh D., Kingsley P.A., Sundar S. (2022). The impact of preoperative oral nutrition supplementation on outcomes in patients undergoing gastrointestinal surgery for cancer in low- and middle-income countries: A systematic review and meta-analysis. Sci. Rep..

[27] Park S.-H., Shin Y.-R., Hur H., Lee C.M., Min J.S., Ryu S.W., Chae H.D., Jeong O., Choi C.-I., Song K.-Y. (2023). Exploring ideal operative time for best outcomes in gastric cancer surgery: A multi-institutional study based on KLASS-07 database. Chin. J. Cancer Res..

[28] https://gco.iarc.who.int/media/globocan/factsheets/cancers/7-stomach-fact-sheet.pdf.

[29] https://gco.iarc.who.int/media/globocan/factsheets/populations/642-romania-fact-sheet.pdf.

[30] Lordick F., Carneiro F., Cascinu S., Fleitas T., Haustermans K., Piessen G., Vogel A., Smyth E. (2022). Gastric cancer: ESMO Clinical Practice Guideline for diagnosis, treatment and follow-up. Ann. Oncol..

[31] Nelen S.D., Verhoeven R.H.A., Lemmens V.E.P.P., de Wilt J.H.W., Bosscha K. (2017). Increasing survival gap between young and elderly gastric cancer patients. Gastric Cancer.

[32] https://gco.iarc.who.int/tomorrow/en/dataviz/bars?multiple_populations=1&mode=cancer&multiple_cancers=1&cancers=7&types=0&populations=8_40_56_70_100_112_191_196_203_208_233_246_250_276_300_348_352_372_380_428_440_442_470_498_499_528_578_616_620_642_643_688_703_705_724_752_756_804_807_826&group_populations=1&years=2050.

[33] Mocan L. (2021). Surgical Management of Gastric Cancer: A Systematic Review. J. Clin. Med..

[34] Ajani J.A., D’Amico T.A., Bentrem D.J., Chao J., Cooke D., Corvera C., Das P., Enzinger P.C., Enzler T., Fanta P. (2022). Gastric cancer, version 2.2022, NCCN clinical practice guidelines in oncology. J. Natl. Compr. Cancer Netw..

[35] Robb W.B., Messager M., Gronnier C., Tessier W., Hec F., Piessen G., Mariette C., FREGAT (French EsoGastric Tumor) Working Group (2015). High-grade toxicity to neoadjuvant treatment for upper gastrointestinal carcinomas: What is the impact on perioperative and oncologic outcomes?. Ann. Surg. Oncol..

[36] Charalampakis N., Xiao L., Lin Q., Elimova E., Shimodaira Y., Harada K., Rogers J.E., Mares J., Amlashi F.G., Minsky B.D. (2017). Co-morbidities rather than age impact outcomes in patients receiving preoperative therapy for gastroesophageal adenocarcinoma. Ann. Surg. Oncol..

[37] Institutul National de Statistica (2021). Recensamantul Populatiei si Locuintelor. Romania. https://www.recensamantromania.ro/rezultate-rpl-2021/rezultate-definitive-caracteristici-demografice/.

[38] Amin M.B., Greene F.L., Edge S.B., Compton C.C., Gershenwald J.E., Brookland R.K., Meyer L., Gress D.M., Byrd D.R., Winchester D.P. (2017). The Eighth Edition AJCC Cancer Staging Manual: Continuing to build a bridge from a population-based to a more “personalized” approach to cancer staging. CA Cancer J. Clin..

[39] Marano L., Verre L., Carbone L., Poto G.E., Fusario D., Venezia D.F., Calomino N., Kaźmierczak-Siedlecka K., Polom K., Marrelli D. (2023). Current Trends in Volume and Surgical Outcomes in Gastric Cancer. J. Clin. Med..

[40] Japanese Gastric Cancer Association (2021). Japanese gastric cancer treatment guidelines 2018 (5th edition). Gastric Cancer.

[41] Degiuli M., De Manzoni G., Di Leo A., D’ugo D., Galasso E., Marrelli D., Petrioli R., Polom K., Roviello F., Santullo F. (2016). Gastric cancer: Current status of lymph node dissection. World J. Gastroenterol..

[42] Marano L., Marrelli D., Roviello F. (2016). Focus on research: Nodal dissection for gastric cancer—A dilemma worthy of King Solomon!. Eur. J. Surg. Oncol. (EJSO).

[43] Sitarz R., Skierucha M., Mielko J., Offerhaus J., Maciejewski R., Polkowski W. (2018). Gastric cancer: Epidemiology, prevention, classification, and treatment. Cancer Manag. Res..

[44] Jensen L., Nielsen H., Mortensen P., Pilegaard H., Johnsen S. (2010). Enforcing centralization for gastric cancer in Denmark. Eur. J. Surg. Oncol. (EJSO).

[45] Dulucq J., Wintringer P., Stabilini C., Solinas L., Perissat J., Mahajna A. (2005). Laparoscopic and open gastric resections for malignant lesions: A prospective comparative study. Surg. Endosc..

[46] Li Q., Zou J., Jia M., Li P., Zhang R., Han J., Huang K., Qiao Y., Xu T., Peng R. (2019). Palliative Gastrectomy and Survival in Patients With Metastatic Gastric Cancer: A Propensity Score–Matched Analysis of a Large Population-Based Study. Clin. Transl. Gastroenterol..

[47] Gertsen E.C., Brenkman H.J., Goense L., Mohammad N.H., Weusten B.L., van Hillegersberg R., Ruurda J.P. (2020). Non-curative gastrectomy for advanced gastric cancer does not result in additional risk of postoperative morbidity compared to curative gastrectomy. Surg. Oncol..

[48] Kopecky K., Monton O., Rosman L., Johnston F. (2022). Palliative interventions for patients with advanced gastric cancer: A systematic review. Chin. Clin. Oncol..

